# Effective omics tools are still lacking for improvement of stress tolerance in polyploid crops

**DOI:** 10.3389/fpls.2023.1295528

**Published:** 2023-10-27

**Authors:** Chao Ding, Zhao Zhang

**Affiliations:** ^1^ Shanxi Center for Testing of Functional Agro-Products, Shanxi Agricultural University, Taiyuan, China; ^2^ Beijing Key Laboratory of Development and Quality Control of Ornamental Crops, Department of Ornamental Horticulture, China Agricultural University, Beijing, China

**Keywords:** abiotic stress, disease resistance, epigenetic regulation, post-transcriptional regulation, phenomics

## Introduction

After decades of development, today’s Omics technologies have advanced tremendously. It is important to note, however, that the vast majority of methodologies in bioinformatics were originally developed for the study of human genetics. Unlike diploid humans, many crop genomes are polyploid, such as tetraploid potato (Sun et al., 2022), hexaploid wheat (Ling et al., 2018; Walkowiak et al., 2020), tetraploid sugarcane (Zhang et al., 2018), octoploid strawberry (Edger et al., 2019), and so on. Existing Omics methods and tools, including genome assembly strategies, algorithm, statistical methods, and efficient data processing and analysis, are far from adequate for the complex genomes of polyploid crops.

## Challenges of genome assembly and annotation

Genome assembly and annotation of polyploid plants is a challenging task due to the complexity of their genomes. Although some new technological tools, such as the highly accurate long-read sequencing technologies, e.g. PacBio HiFi (Hon et al., 2020), have made it possible to accurately obtain genomic fragments that distinguish haplotypes. However, the presence of highly similar sequences tends to make assembly of repetitive regions difficult. Therefore, obtaining haplotype-resolved polyploid genomes of high quality is still a difficult task. It is worth noting that compared with allopolyploid plants, this issue is more prominent for autopolyploid plants. People have attempted to assemble the chromosome-level genome of polyploid plants using High-throughput chromosome conformation capture (Hi-C) technology (Dekker, 2006) and ALLHiC software (Zhang et al., 2019), but this approach fails in autopolyploid plants in many cases (Bao et al., 2022; Sun et al., 2022). Due to the lack of reliable bioinformatic methods, obtaining high-quality autopolyploid plant genomes can only be achieved through experimental methods. For example, the genome of autotetraploid potato was obtained by sequencing a total of 1034 individuals from a selfing population (F2 population; Bao et al., 2022), which obviously involves significant time and cost.

Furthermore, this challenge becomes even more pronounced for crops with highly heterozygous genomes. The presence of multiple alleles and variations within the genome adds an extra layer of complexity to the assembly and annotation processes. Resolving the allelic diversity and accurately assigning variations to specific loci can be particularly challenging in highly heterozygous crops. Advanced computational algorithms and innovative sequencing technologies are continually being developed to address these complexities and improve the accuracy of genome assembly and annotation in highly heterozygous crops.

## Complex genome structure

Polyploid plants typically possess large genomes, e.g. approximately 16 Gb for wheat (Walkowiak et al., 2020) and 7.68 Gb for tetraploid rhubarb (*Rheum officinale*, a perennial medicinal herb) (Zhang et al., 2023a). For the crops that rely mainly on asexual propagation (many of which are horticultural), e.g. the tetraploid modern rose (*Rosa hybrida*), their genomes maintain a high level of heterozygosity due to a lack of sexual reproduction (Zhang et al., 2023b). The presence of multiple homologous copies (alleles) in the genome increases complexity to the analysis and interpretation of genomic information. Distinguishing different alleles and resolving dosage effect of gene can be challenging. Different studies have found that the expression between alleles in polyploid plants can vary significantly or may differ only slightly (Zhang et al., 2023b).

## Complexity of epigenetic regulation and post-transcriptional regulation

In addition to genomic and transcriptomic complexity, polyploid plants have complex epigenetic mechanisms. Epigenetic regulation involves dynamic modifications of nucleic acids and histones, encompassing both genomic and transcriptional levels (Shen et al., 2017). Additionally, non-coding RNAs, such as miRNA, siRNA, piRNA and long non-coding RNAs, are involved in the epigenetic regulation of gene expression by targeting mRNA stability and translation (Shi et al., 2022). Epigenetic modifications may have important effects on gene expression in plants, but the complexity of the epigenetic regulatory network makes it difficult to accurately understand and interpret (Ma et al., 2015). The complexity of epigenetic regulation lies in its multilevel nature, involving interactions among DNA methylation (and methylome), histone modifications, chromatin remodeling, noncoding RNAs, and various regulatory proteins. Moreover, the three-dimensional organization of chromatin within the nucleus, refers as 3D genome, have significant implications for gene regulation and other cellular processes. Understanding the 3D genome and its dynamics provides insights into how genetic information is regulated spatially and temporally, impacting processes like plant growth, development, stress responses and so on. The interactions between different subgenomes in polyploid plants undoubtedly add another layer of complexity of their epigenetic regulation.

## Reliable phenomics is necessary for effective interpretation of complex traits

In most cases, stress tolerance in plants is a complex trait. This applies not only to abiotic stress tolerance but also to resistance against biotic stress. Plant complex traits are typically determined by multiple genes and their interactions. These genes may be located on different chromosomes, making direct linkage analysis challenging, and polyploidy further exacerbates this complexity. Moreover, the effects of these genes are often subtle, resulting in minimal phenotypic differences from changes in a single gene. Therefore, reliable and high-throughput phenomics is required. Accurate assessment of phenotypes is essential for effective genetic analysis.

## Data analysis require appropriate statistical models and efficient computation

The extensive data generated from polyploid plant genomics research requires complex analysis and interpretation. Challenges include standardized data processing, establishing effective analysis pipelines, and developing suitable statistical models. Additionally, integrating genomic data with phenotype information poses a further challenge in gaining deeper insights into the characteristics of polyploid plants. These tasks often rely on powerful parallel computational capabilities, and thus, developing efficient computations based on Graphics Processing Units (GPUs) can significantly enhance performance.

## Prospects

**Figure 1:**
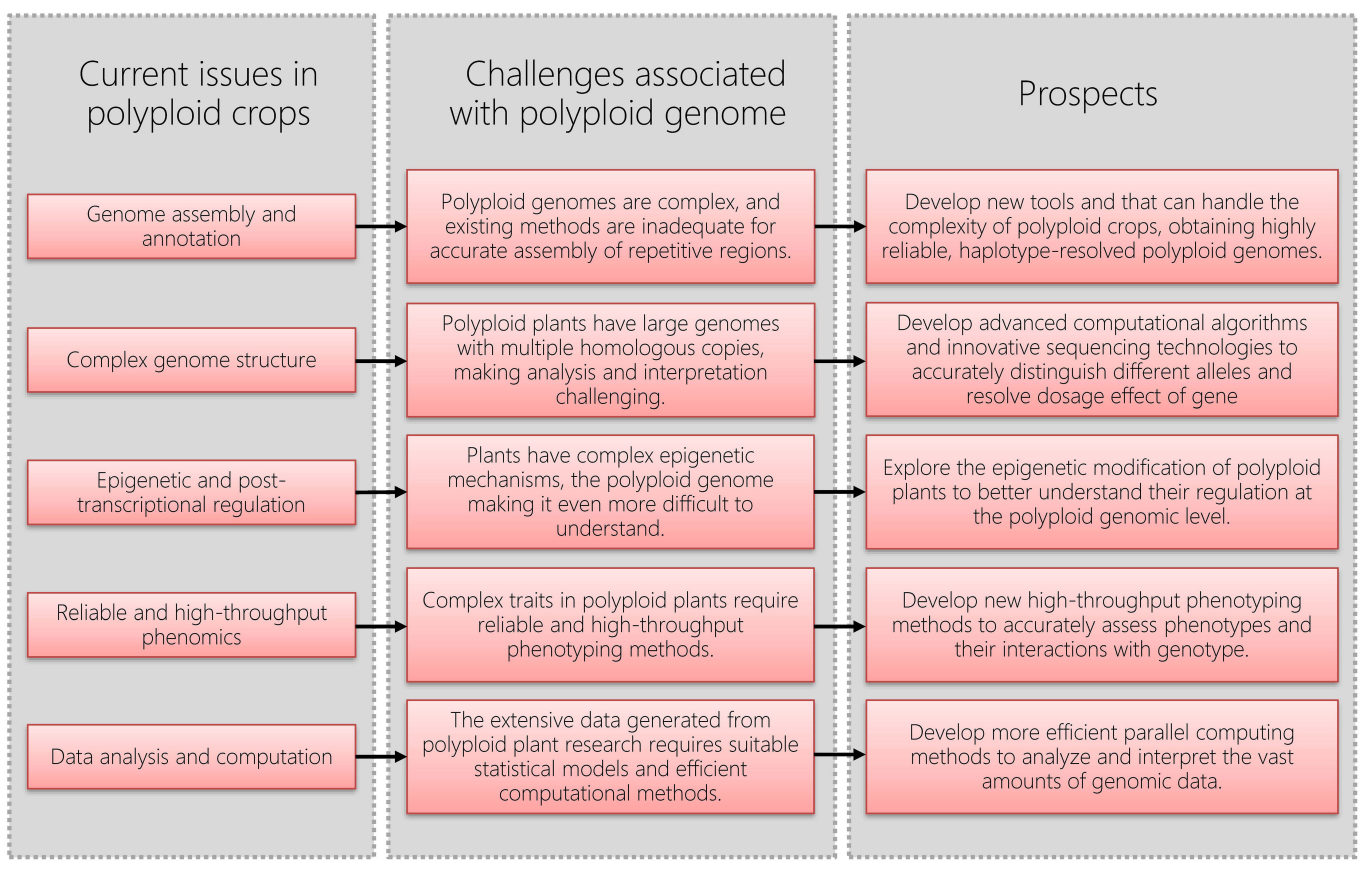
Challenges and prospects in polyploid crop genome research.

In the past decade, many software tools applicable to polyploids have been developed, such as fitTetra (Voorrips et al., 2011; Zych et al., 2019) and polymapR (Bourke et al., 2018). However, genomic research on polyploid plants still faces challenges regarding complex genome structure, genome assembly and annotation, the complexity of epigenetic regulation, as well as the complexity of data analysis and interpretation (**Figure 1**). Overcoming these challenges requires continuous technological development and innovation, as well as enhanced collaboration and communication across different disciplines, to further advance genomic research on polyploid plants.

## Author contributions

CD: Writing – original draft, Writing – review & editing. ZZ: Writing – original draft, Writing – review & editing.
